# An Important Role of the Type VI Secretion System of Pseudomonas aeruginosa Regulated by Dnr in Response to Anaerobic Environments

**DOI:** 10.1128/spectrum.01533-22

**Published:** 2022-10-27

**Authors:** Jing Dang, Tietao Wang, Jing Wen, Haihua Liang

**Affiliations:** a Key Laboratory of Resources Biology and Biotechnology in Western China, Ministry of Education, College of Life Sciences, Northwest University, Xi'an, Shaanxi, China; Griffith University

**Keywords:** *Pseudomonas aeruginosa*, Dnr, H2-T6SS, ModA, anaerobic condition

## Abstract

The type VI secretion system (T6SS) is capable of secreting a variety of metal-binding proteins involved in metal ion uptake, and it mediates an active metal ion transport system that contributes to competition between bacteria. Pseudomonas aeruginosa H2-T6SS can increase molybdenum ion acquisition and enhance bacterial survival advantage by promoting the secretion of the molybdate-binding protein ModA, in which the expression of H2-T6SS core genes *hcp2*, *hsiA2*, and *clpV2* is activated by anaerobic conditions and are all regulated by the global regulator Anr. Here, we report the regulation of T6SS by Dnr, a dedicated dissimilatory nitrate respiration regulator in P. aeruginosa. Of the three distinct T6SS loci carried by P. aeruginosa, only the anaerobic expression of H2-T6SS was activated by Dnr; H1-T6SS or H3-T6SS did not respond to anaerobically induced activation. We also demonstrated that Dnr promotes the anaerobic secretion of ModA, which acts as a potential substrate for H2-T6SS, providing an advantage not only for the anaerobic growth of bacteria but also for functional competition. Overall, this study elucidates the important role played by Dnr in mediating the anaerobic expression of T6SS in P. aeruginosa, indicating that the functional advantage of H2-T6SS in response to anaerobic induction may be a conditional environmental adaptation. It also extends our understanding of the function of Dnr as a specific regulator of dissimilatory nitrate respiration.

**IMPORTANCE** The type VI secretion system (T6SS) plays an important role in bacterial competition by mediating the transport of active metal ions. Pseudomonas aeruginosa carries three distinct T6SS loci (H1-, H2-, and H3-T6SS). The H2-T6SS promotes the secretion of the molybdate-binding protein ModA for the acquisition of molybdenum ions to adapt to anaerobic survival. Here, we report that the specialized dissimilatory nitrate respiration regulator Dnr in P. aeruginosa controls the anaerobic expression of H2-T6SS and that this regulation is essential for ModA protein secretion, anaerobic growth, and bacterial competition. This study elucidates the regulatory mechanism of Dnr on H2-T6SS in P. aeruginosa, revealing an important role played by H2-T6SS in adapting to an anaerobic environment.

## INTRODUCTION

The Gram-negative bacterium Pseudomonas aeruginosa is the main pathogen causing chronic lung infections in cystic fibrosis (CF) patients (1). During chronic CF infection, P. aeruginosa strains transform into a mucus phenotype, which is driven by the CF microenvironment and exhibits upregulated alginate production to facilitate the formation of biofilm (1, 2). Biofilms are an important form of growth for P. aeruginosa to survive under unfavorable conditions (3). Worlitzsch et al. demonstrated that P. aeruginosa biofilms in CF lungs grew in stagnant mucus, an environment in which anaerobic conditions prevailed (4). Biofilms formed under anaerobic conditions were thicker and denser than the small colonies typical of biofilms formed under aerobic conditions (3), and this robust biofilm acted as a diffusion barrier, restricting the entry of antibiotics into bacterial cells and producing multidrug-resistant persistent cells that are also responsible for long-term and recurrent infections in CF patients (5, 6), demonstrating the highly adaptive nature of anaerobic growth pattern of P. aeruginosa. Therefore, research into the pathogenesis and treatment of CF should include a better understanding of the anaerobic metabolism of P. aeruginosa.

The growth of P. aeruginosa under anaerobic conditions relies primarily on the nitrate respiration pathway (7, 8). In this process, nitrate (NO_3_^−^) replaces molecular oxygen (O_2_) as the terminal electron acceptor, involving a continuous eight-electron dissimilatory reduction from NO_3_^−^ to N_2_, also known as denitrification (7, 9). Each step of the bacterial denitrification pathway is catalyzed by a single metalloenzyme, such as nitrate reductase (NAR), nitrite reductase (NIR), nitric oxide reductase (NOR), and nitrous oxide reductase (N_2_OR) (10, 11). In P. aeruginosa, Anr (an anaerobic regulatory protein of arginine deiminase and nitrate reductase) and Dnr (a dissimilatory nitrate respiration regulatory protein) are two important anaerobic transcriptional regulators that initiate the expression of a range of reductases in the nitrate respiration pathway (12). However, Anr directly controls the expression of NAR; it does not directly regulate the activity of NIR, NOR, and N_2_OR in the denitrification pathway. Anr indirectly regulates the expression of reductases associated with the pathway by activating the expression of NarXL, a two-component nitrate-sensing regulatory system, and by controlling the expression of Dnr (13). Indeed, Anr is a global anaerobic regulator and Anr-dependent regulation of arginine deiminase activity and cyanide production systems is not regulated by Dnr, which is dedicated to nitrate respiration and is required for the expression of all four structural genes of denitrifying enzymes (12–14). Anr is not required for denitrification when the transcriptional regulator Dnr is compulsorily expressed. The control of Dnr transcription by Anr demonstrates that the Anr-dependent induction of the denitrification pathway is a secondary effect of Dnr expression (12, 13). As a consequence, Dnr is specifically in activating the entire denitrification pathway in P. aeruginosa.

P. aeruginosa can survive under a wide range of environmental conditions, relying on its high degree of adaptability to the environment, and continuously infect its hosts (15, 16). The pathogenicity of P. aeruginosa relies on its complex secretion system, of which the type VI secretion system (T6SS) is essential in chronic infections (17, 18). Three evolutionarily distinct groups of T6SS motifs exist in P. aeruginosa, namely, H1-T6SS, H2-T6SS, and H3-T6SS, each with ~15 to 20 genes with distinct structural components and functions (17, 19–22). T6SS delivers effectors to bacterial targets in a contact-independent manner and is involved in the acquisition of metal ions (23–25). Metal ions (such as iron, copper, and molybdenum) are components and signals of enzymes, regulatory proteins, and protein complexes involved in metabolism during cellular life. In P. aeruginosa, the TseF protein secreted by H3-T6SS is involved in iron uptake by interacting with the outer membrane vesicle (OMV) and the Pseudomonas quinolone signaling (PQS) system, and due to its unique redox potential, iron plays an important role as a cofactor for many enzymes essential for cellular metabolic processes (23). A Cu^2+^-binding protein, Azu, is a substrate for P. aeruginosa H2-T6SS. H2-T6SS facilitates the secretion of Azu, which mediates the uptake of extracellular Cu^2+^. Azu interacts with the outer membrane transporter protein OprC and is secreted to other bacteria to maintain cellular copper ion homeostasis and enable the bacteria to adapt to different environments (26). The molybdate-binding protein ModA in P. aeruginosa is dependent on H2-T6SS for secretion and is involved in the transport of molybdenum ions through the mediation of the outer membrane receptor protein IcmP to adapt to competition and growth in anaerobic environments (27). As described above, T6SS is indispensably required to promote the secretion of metal ion-related binding proteins to mediate active ion transport, thereby enhancing the environmental adaptability of bacteria.

Three T6SS loci (H1-, H2-, and H3-T6SS) have been identified in P. aeruginosa (17). Here, we report that under anaerobic conditions, the expression of H2-T6SS is promoted by the transcriptional regulator Dnr. The secretion of the molybdate-binding protein ModA is dependent on H2-T6SS (27). In conjunction with the present study, we also found that Dnr promotes anaerobic secretion of the ModA protein, which provides an advantage for anaerobic growth and competition in bacteria. These findings highlight the functional advantage of H2-T6SS in response to anaerobic induction and extend our understanding of the role of Dnr as a specific regulator of nitrate respiration.

## RESULTS

### Dnr activates the expression of H2-T6SS under anaerobic conditions.

**FIG 1:**
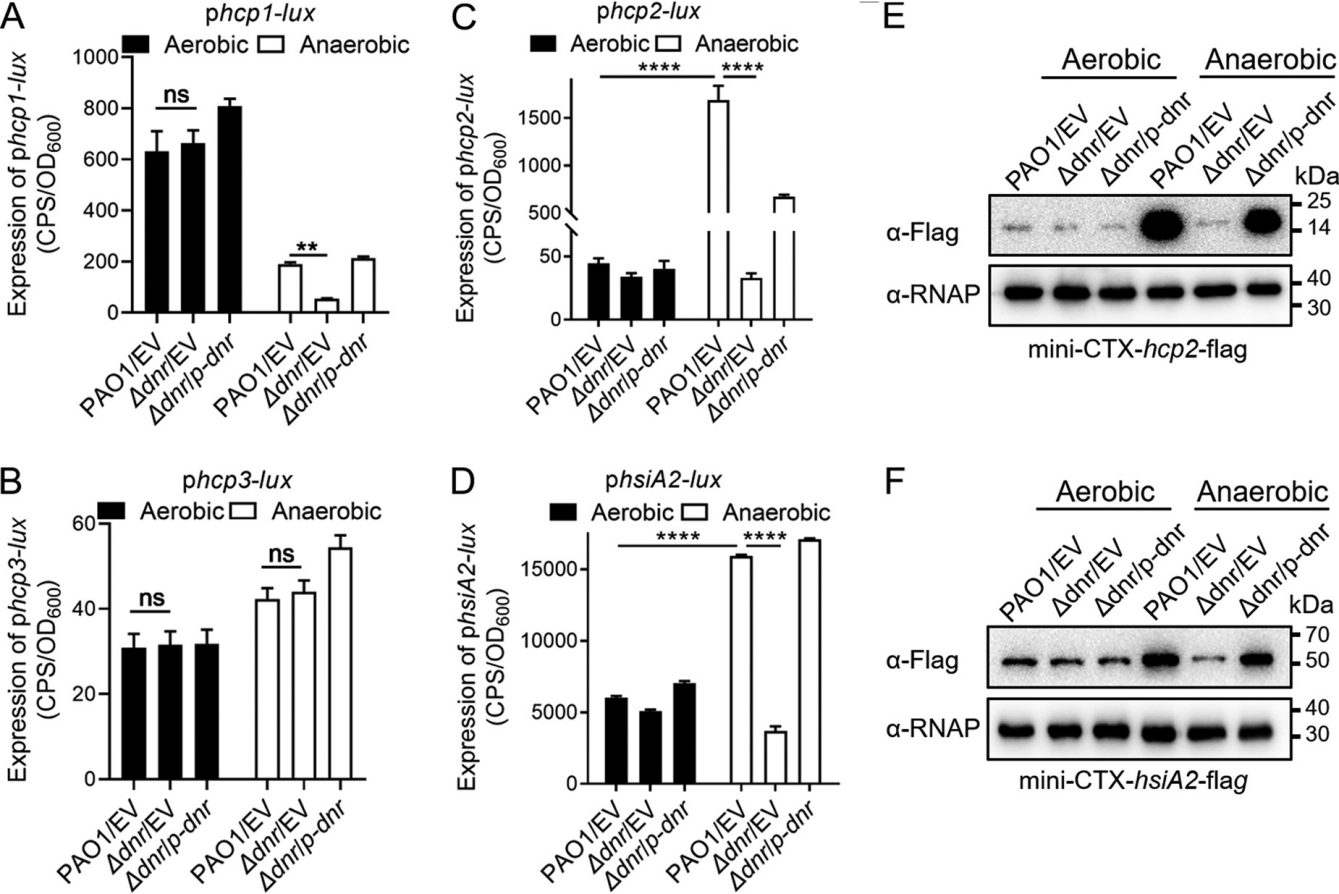
Dnr activates the expression of H2-T6SS under anaerobic conditions. (A to D) *lux*-based reporter analysis of wild-type P. aeruginosa, the Δ*dnr* mutant, and the complemented strains harboring *hcp1* (A), *hcp3* (B), *hcp2* (C), or *hsiA2* (D) *lux* reporters were cultured under aerobic or anaerobic conditions to an OD_600_ of 1.0 in LB containing 15 mM KNO_3_. The data shown are the average from three independent experiments; error bars indicate standard deviation (SD) from three independent experiments. Statistical significance was calculated using two-way ANOVA with Dunnett′s multiple-comparison tests. ****, *P* < 0.0001; **, *P* < 0.01; ns, not significant. (E and F) Western blot analysis of wild-type P. aeruginosa, the Δ*dnr* mutant, and the complemented strains harboring *hcp2* (E) or *hsiA2* (F) with Flag probe was cultured under aerobic or anaerobic conditions to an OD_600_ of 1.0 in LB containing 15 mM KNO_3_. Similar results were obtained from three independent experiments, and the data shown are from one representative experiment.

Dnr is involved in the transcriptional activation of dissimilatory nitrate respiration (8, 12–14, 28, 29). To explore whether the gene expression of T6SS is affected by Dnr, we first used the gene knockout method of homologous recombination to construct a *dnr* gene-deficient (Δ*dnr*) strain successfully. Next, we used the related promoter-*lux* reporters and measured their activities in the wild-type PAO1 strain, the Δ*dnr* strain, and its complemented (Δ*dnr/p-dnr*) strain under aerobic and anaerobic conditions, respectively. The hemolysin-coregulated protein Hcp is the basis for exploring T6SS (18, 30). *hsiA2* (*PA1656*) is the first gene in the H2-T6SS gene cluster, which initiates the transcriptional regulation mode of this cluster (20). As shown in Fig. 1, the transcriptional levels of Hcp2 and HsiA2 were much higher when strains were cultured under anaerobic conditions than under aerobic conditions (Fig. 1C and D). However, the level of Hcp1 transcription was decreased under anaerobic conditions (Fig. 1A). In addition, there was no significant difference in the transcriptional level of Hcp3 under aerobic conditions from that under anaerobic conditions (Fig. 1B). In the Δ*dnr* mutant, the transcriptional levels of Hcp1, Hcp2, and HsiA2 were significantly reduced compared with those of the wild-type PAO1 under anaerobic conditions, indicating that Dnr showed activity under anaerobic conditions and affected the transcriptional expressions of H1-T6SS and H2-T6SS. At the moment, it is worth noting that the luminescence of Hcp1 was reduced in Δ*dnr* mutant, but the expression in the wild-type PAO1 strain has been inhibited under anaerobic conditions. This suggested that the anaerobic expression of Hcp1 was affected by Dnr positively; however, it might be simultaneously controlled through other anaerobic condition-related genes, not only by Dnr. The levels of Hcp2 and HsiA2 decreased significantly due to the deletion of *dnr* under anaerobic conditions (Fig. 1C and D), indicating that Dnr is critical for the transcriptional expression of H2-T6SS.

Dnr is an anaerobic transcriptional regulator. Based on the above results, we will focus on exploring the regulation of Dnr on H2-T6SS under anaerobic conditions. The mini-CTX-*lacZ* plasmid expressed by the chimeras of *hcp2*-Flag and *hsiA2*-Flag were integrated into the wild-type PAO1 strain, the Δ*dnr* strain, and its complemented (Δ*dnr*/*p-dnr*) strain to construct the fusion strains, respectively. Western blot assay detected the regulation of Dnr on the protein expression level of H2-T6SS. The results showed that the protein expression of Hcp2 or HsiA2 was upregulated under anaerobic conditions in PAO1, but in the Δ*dnr* mutant, expression was significantly reduced (Fig. 1E and F). This indicated that Dnr was a positive regulator for expressing the protein of H2-T6SS under anaerobic conditions.

### Dnr binds to the promoter regions of H2-T6SS genes.

**FIG 2:**
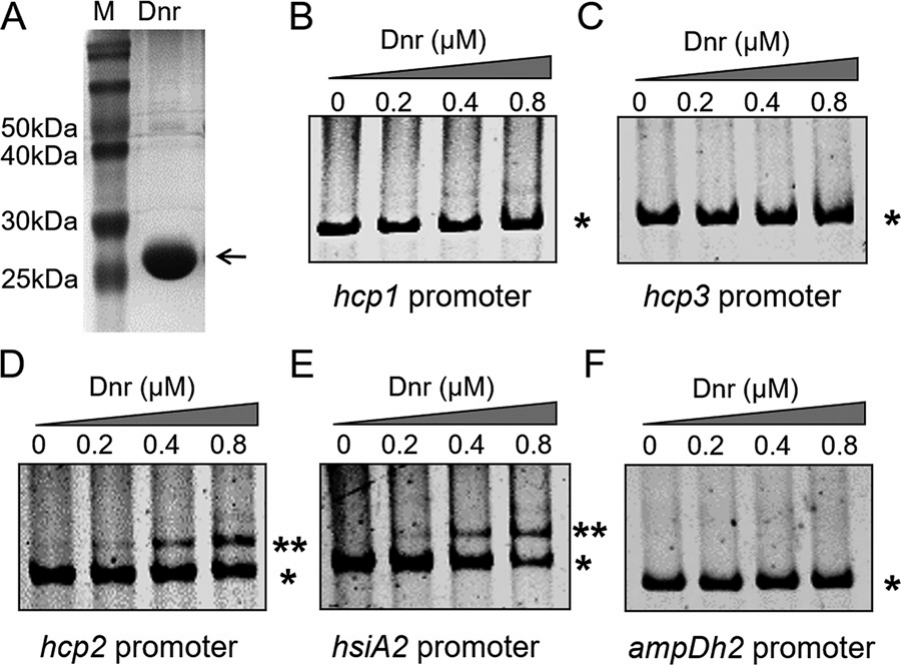
Dnr binds to the promoter regions of H2-T6SS genes. (A) High-purity target protein Dnr (~26 kDa). M represents the prestained protein indicator marker. (B to F) Dnr binds to the H2-T6SS promoter regions. PCR products containing *hcp1* (B), *hcp3* (C), *hcp2* (D), *hsiA2* (E), and *ampDh2* (F) promoter fragments were added to the reaction mixture at a concentration of 1.5 ng. Promoter *ampDh2* (F) served as a negative control. The protein concentration of each sample is indicated above the lanes. *, free promoter; **, protein-promoter complex. Similar results were obtained from three independent experiments, and the data shown are from one representative experiment.

The *dnr* gene encodes a protein of 227 amino acids, and the molecular weight of the gene product is about 26 kDa (14). To determine whether the influence of Dnr on T6SS expression was the result of direct binding of the transcriptional regulator to these promoters, an electrophoretic mobility shift assay (EMSA) was performed using the T6SS promoter regions (see Fig. S1 in the supplemental material) and purified Dnr protein (Fig. 2A). Promoter probes containing *hcp1*, *hcp2*, *hcp3*, or *hsiA2* were reacted with the purified Dnr protein, respectively, and the irrelevant promoter *ampDh2* was used as a negative control. In contrast to the negative control, the incubation of a probe containing *hcp2* or *hsiA2* promoter fragments with Dnr protein led to the formation of DNA-protein complexes (Fig. 2D to F). Compared to *hcp2* and *hsiA2*, neither the *hcp1* nor *hcp3* DNA fragment was bound by Dnr obviously (Fig. 2B and C). This suggests that Dnr regulates H2-T6SS activity by directly binding to the *hcp2* and *hsiA2* promoter regions.

### Dnr facilities the activity of the H2-T6SS injection apparatus by activating the expression of ClpV2 under anaerobic conditions.

**FIG 3:**
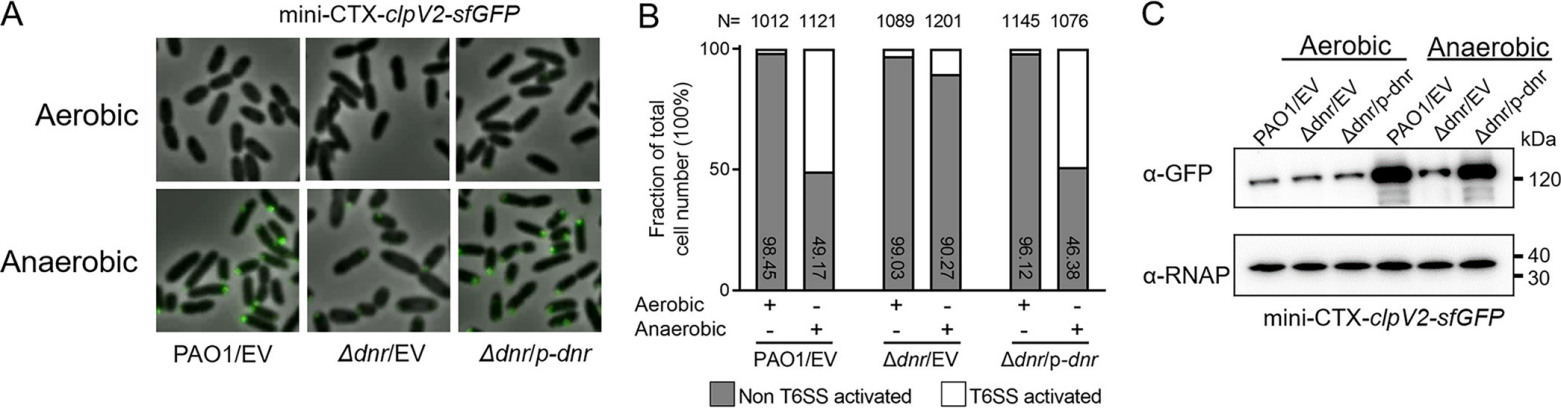
H2-T6SS expression and its assembly are activated under anaerobic conditions via Dnr. (A and B) *clpV2-sf*GFP localization in the wild-type P. aeruginosa, the Δ*dnr* mutant, and the complemented strains was measured by fluorescence microscopy. Cells were cultured under aerobic or anaerobic conditions. (C) Western blot analysis of wild-type P. aeruginosa, the Δ*dnr* mutant, and the complemented strains harboring *clpV2*-*sfGFP* cultured under aerobic or anaerobic conditions to an OD_600_ of 1.0 in LB containing 15 mM KNO_3_. Similar results were obtained from three independent experiments, and the data shown are from one representative experiment.

The secretion of P. aeruginosa T6SS requires the assistance of structural proteins (31, 32). As a typical structural gene, *clpV* is indispensable for the function of T6SS (20, 33). The contracted sheath of the T6SS injection apparatus in P. aeruginosa is specifically recognized and disassembled in a ClpV-dependent manner to release subunits and thus recycle them for a new round of an extended sheath reassembly (34, 35). ClpV2 is an energetic driver of the secretion apparatus of H2-T6SS (20, 33). To further determine the regulation of protein level of H2-T6SS by Dnr, we tested the expression of the core gene *clpV2 in vivo*. The *clpV2*-*sf*GFP fusion (expressing green fluorescent protein [GFP]) was located in the related strains of P. aeruginosa, and the protein expression of ClpV2 was directly detected in the cell by fluorescence microscopy assay. Our results showed that there were no visible fluorescent foci in the related strains of P. aeruginosa under aerobic conditions. Under anaerobic conditions, the fluorescent foci were significantly increased in wild-type PAO1 but dramatically decreased in the Δ*dnr* mutant (Fig. 3A and Video S1). The absence of *dnr* reduced the number of cells with visible ClpV2-sfGFP foci by more than 50% compared to the wild-type strain under anaerobic conditions (Fig. 3B). At the same time, the Western blot assay was used as a reference under the same experimental conditions, and the same results were verified *in vivo* and *in vitro* (Fig. 3C). These results showed that Dnr activates the protein expression of the H2-T6SS structural gene *clpV2* by anaerobiosis, which can promote the disassembly and secretion of the T6SS injection apparatus.

### Both Dnr and H2-T6SS are required for nitrate respiration and anaerobic growth.

**FIG 4:**
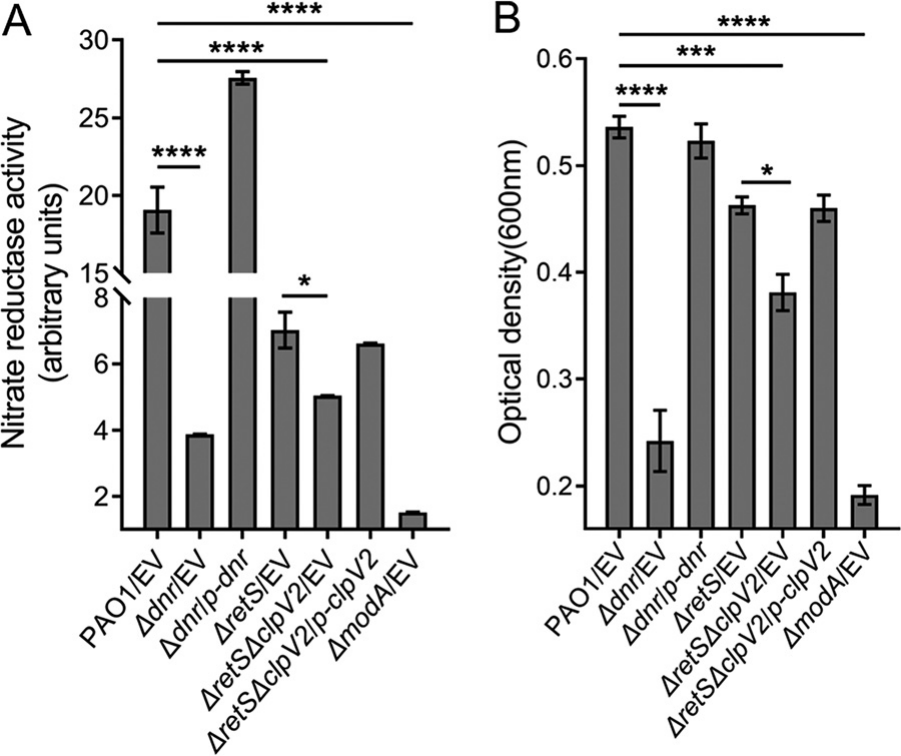
Dnr and H2-T6SS are important for P. aeruginosa growth under anaerobic conditions. Wild-type P. aeruginosa and the derivative mutant strains were detected in LB containing 15 mM KNO_3_ at 37°C for 24 h under anaerobic conditions, and nitrate reductase activity (A) and optical density (B) were detected. (A and B) The data shown are the average from three independent experiments; error bars indicate the SD from three independent experiments. Statistical significance was calculated using one-way ANOVA with Dunnett′s multiple-comparison test. ****, *P* < 0.0001; ***, *P* < 0.001; *, *P* < 0.05.

Dnr is required for the expression of the structural genes of all four metalloenzymes that catalyze the bacterial denitrification pathway under hypoxic or anaerobic conditions (12, 13, 29). Above, we confirmed that Dnr exactly activates the expression of ClpV2 under anaerobic conditions (Fig. 3). Therefore, we speculated that the reduced expression of ClpV2 caused by the absence of *dnr* would also affect the nitrate reduction pathway, which not only demonstrates that the presence of Dnr and ClpV2 provides the bacteria with intact anaerobic growth but also confers on P. aeruginosa an advantageous function in an anaerobic environment because T6SS is a versatile protein export machinery capable of translocating effectors into target cells (19, 23, 26, 27, 34, 36). Nitrate reductase required for the anaerobic growth of P. aeruginosa requires the molybdenum cofactor (MoCo) to activate the enzyme activity (13). Molybdenum (Mo) is incorporated into MoCo and is essential for the activity of molybdenum enzymes (10). Molybdate (MoO_4_^2−^) is the usable form of the trace element Mo (37). ModA, a molybdate-binding periplasmic protein precursor with a high affinity for MoO_4_^2−^, is responsible for the extracellular uptake of MoO_4_^2−^ (38, 39). Mutant strains lacking *modA* eliminated the accumulation of Mo and thus were unable to utilize nitrate for anaerobic respiration in basal medium (10, 39). In a previous study, we found that ModA in P. aeruginosa is an effective substrate for H2-T6SS. For this purpose, we used the Δ*modA* strain as a reference and compared the nitrate reduction rates of the Δ*dnr* and Δ*clpV2* strains under anaerobic conditions. The nitrate reductase activity of the Δ*modA* mutant was reduced by ~90% compared to the wild-type parent (Fig. 4A), which is consistent with previous studies (27, 39). The deletion of *dnr* under anaerobic conditions significantly reduced the activity of nitrate reductase by 79.6%, and the enzyme activity was restored after the *dnr* gene was complemented (Fig. 4A). This suggests that the Δ*dnr* mutant is essentially close to the level of inhibition of the Δ*modA* mutant. RetS is a signal transduction sensor that strongly represses the expression of H2-T6SS through the GacS/GacA and Rsm signaling pathways (36, 40). The Δ*retS* mutant shows lower nitrate reductase activity than the wild-type parent (27). We found that the deletion of *clpV2* in the context of the *retS* mutation resulted in lower enzyme activity by 28.1% than in Δ*retS* mutant (Fig. 4A). (The Δ*clpV2* single mutation shows lower nitrate reductase activity by 10.4% than that in the wild-type parent [data not presented here].) These findings indicate that both Dnr and H2-T6SS are required for the regulation of reductase activity.

Bacteria utilize nitrate reductase to catalyze nitrate reduction for anaerobic respiration (41). Following the deletion of either *modA*, *dnr*, or *clpV2*, which reduced reductase activity, we monitored the anaerobic growth rates. The 90% reduction in reductase activity in the Δ*modA* mutant resulted in ~65% lower growth. Compared to the wild-type parent, the growth of the Δ*dnr* mutant under anaerobic conditions declined by 54.8%. In the Δ*retS* background, the deletion of *clpV2* resulted in a 17.7% reduction in growth rate (Fig. 4B). These results suggest that not only ModA but also Dnr and H2-T6SS are essential for the survival of P. aeruginosa in anaerobic environments. Here, since the nitrate reduction and anaerobic growth in P. aeruginosa are Mo dependent (13, 39), Dnr directly controls the expression of NAR in the denitrification pathway (12, 13, 29), and its effect on molybdenum ion acquisition can be directly confirmed by the eventual alteration of molybdenum enzyme activity and the resulting attenuation of growth rate, we did not specifically seek to measure molybdate concentrations in Δ*dnr* cells. The reduction in nitrate reductase activity and anaerobic growth caused by the mutation should be more pronounced than the results measured in the present experiments, as a short period is required for the growth chamber to become anaerobic.

### Dnr promotes the secretion of ModA, which contributes to a competitive advantage for bacteria under anaerobic conditions.

**FIG 5:**
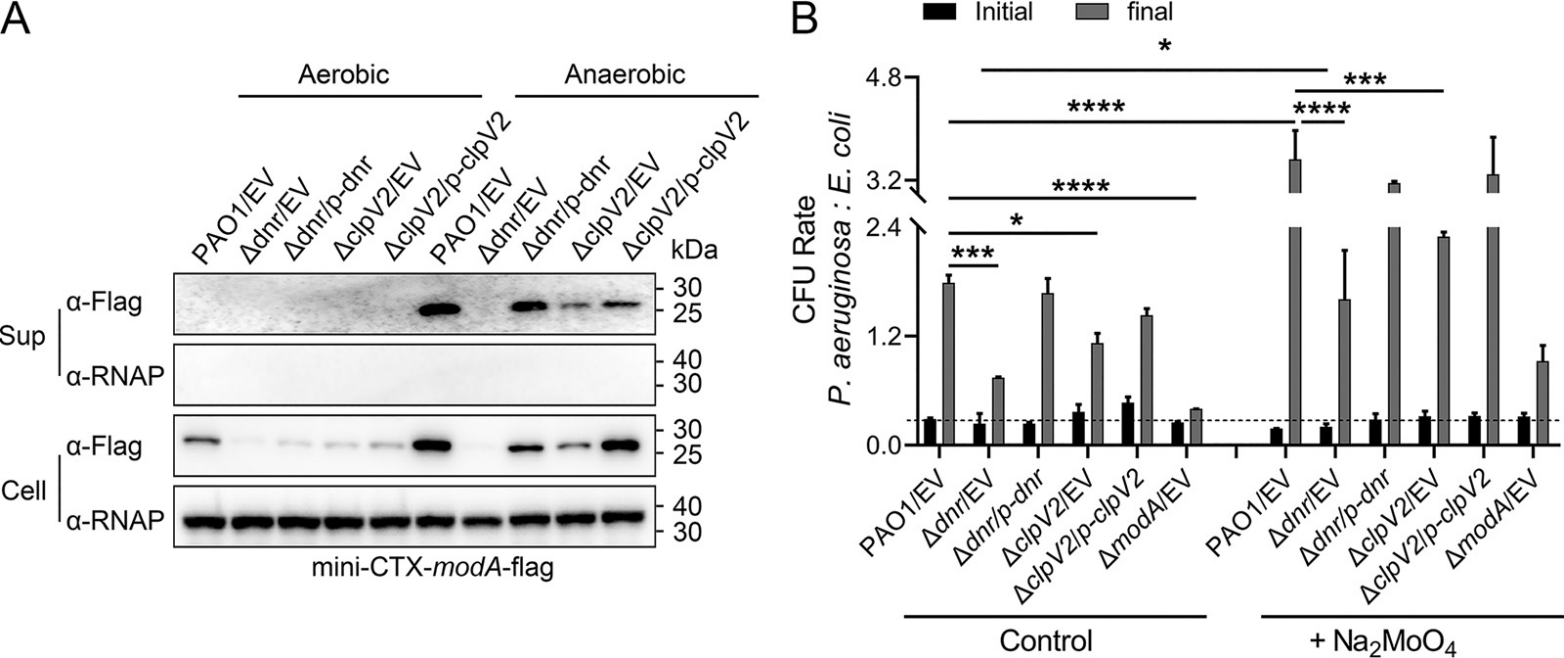
(A) ModA secretion is activated by Dnr during anaerobic conditions. The *modA*-Flag expression plasmid mini-CTX-*modA*-flag was transformed into each of the wild-type P. aeruginosa, Δ*dnr* mutant, Δ*clpV2* mutant, and complemented strains, which were cultured under aerobic or anaerobic conditions to an OD_600_ of 1.0 in LB containing 15 mM KNO_3_. Western blot analysis of *modA*-Flag in the cell (Cell) and concentrated supernatant (Sup) protein fractions from the indicated strains. For the cell fraction, an antibody against RNA polymerase α (RNAP) was used as the loading control. Similar results were obtained from three independent experiments, and the data shown are from one representative experiment. (B) H2-T6SS and Dnr provide a growth advantage for PAO1 during interbacterial competition in an anaerobic environment. Shown are the results from interbacterial growth competition assays between P. aeruginosa and E. coli. Competition between the indicated strains was examined in M9 containing 15 mM KNO_3_ at 37°C for 12 h under anaerobic conditions. Quantification of CFU before (initial) and after (final) growth competition assays between the indicated organisms was performed. The CFU ratio of the relevant P. aeruginosa strains versus the competitors is plotted. The data shown are the average of three independent experiments; error bars indicate the SD from three independent experiments. Statistical significance was calculated using one-way ANOVA with Dunnett′s multiple-comparison test. ****, *P* < 0.0001; ***, *P* < 0.001; *, *P* < 0.05.

The secretion of ModA protein to the extracellular surface of bacteria is important for the anaerobic pathogenic mechanism of P. aeruginosa (10, 19). It has been demonstrated that the secretion of ModA in P. aeruginosa is dependent on H2-T6SS and is mediated by the outer membrane receptor protein IcmP, which is involved in the translocation of molybdenum ions to adapt to competition and growth in an anaerobic environment (27). We also found as described above that the transcriptional regulator Dnr activates the expression of H2-T6SS under anaerobic conditions. Then, considering whether Dnr affects the secretion of ModA, we used the mini-CTX-*modA*-flag expression plasmid to detect the amount of ModA protein in bacterial culture supernatants under anaerobic conditions via Western blotting. The results showed that ModA protein secretion was higher under anaerobic conditions than aerobic conditions, but it was significantly lower in the Δ*dnr* mutant. When the deletion of *dnr* was complemented, ModA protein secretion was restored to wild-type levels (Fig. 5A). The level of the secretion of ModA in Δ*clpV2* mutant was also lower than wild-type levels, consistent with the previous identification of ModA as a substrate for ClpV2 (27), but not as significantly as in the Δ*dnr* mutant (Fig. 5A). These findings confirmed that Dnr promotes the secretion of ModA under anaerobic conditions, and the effect may rely on the activation of ClpV2 by Dnr.

The absence of *modA* is a less competitive advantage than P. aeruginosa wild-type PAO1 has under anaerobic growth (10). Therefore, we used the Δ*modA* mutant as a reference to assess the importance of Dnr and ClpV2 in mediating interbacterial growth competition under anaerobic conditions. Strains lacking *dnr* or *clpV2* reduced the interspecies competitive advantage of the P. aeruginosa wild type against Escherichia coli, and the reduction in competitive ability caused by the Δ*dnr* mutation was more pronounced than that caused by Δ*clpV2* (Fig. 5B and Fig. S2B). Furthermore, the competitive advantage of wild-type PAO1 was greatly improved under 10 μM MoO_4_^2−^ conditions, whereas it was slight for the mutant strains (Fig. 5B and Fig. S2B). (The micromolar concentration of MoO_4_^2−^ was chosen as described in previous studies [27]). Altogether, these data indicate that similar to ModA, Dnr, and ClpV2 are indispensable for bacterial growth competition in anaerobic environments.

## DISCUSSION

Known as a “molecular weapon,” the T6SS is capable of secreting a variety of metal-binding proteins involved in metal ion acquisition, and the active metal ion transport system mediated by T6SS plays an important role in contact-independent bacterium-to-bacterium competition (23, 24, 26, 27). Metal ions (such as iron, copper, and molybdenum) are components and signals of enzymes, regulatory proteins, and protein complexes involved in metabolism in cellular life processes (23, 26, 38). Recent studies have shown that P. aeruginosa H2-T6SS can increase the acquisition of molybdenum ions and enhance the survival advantage of bacteria by promoting the secretion of the molybdate-binding protein ModA (27). In this work, we show that the dissimilatory nitrate respiration regulator Dnr in P. aeruginosa encourages the anaerobic expression of H2-T6SS and that this regulation is essential for ModA protein secretion, anaerobic growth, and bacterial competition. These findings demonstrate the functional advantage of H2-T6SS in response to anaerobic induction.

Anr, a global anaerobic regulator, belongs to the family of anaerobic transcriptional regulators together with Dnr (14, 29, 42–47). In the denitrification pathway, the expression of Dnr is controlled by Anr (8, 12, 13). In previous studies, we found that the expression of genes of the multifunctional T6SS under anaerobic conditions is mediated by Anr (27). It has been confirmed that Anr activates the anaerobic expression of H2-T6SS structural genes *hcp2* and *hsiA2*, whereas the anaerobic expression of H1-T6SS and H3-T6SS has not been directly proved. Here, we investigated the regulation of the three groups of T6SS structural genes *hcp* by the specific nitrate respiration regulator Dnr and found that Dnr activated the expression of Hcp1 and Hcp2 under anaerobic conditions, while the transcriptional expression of Hcp3 was not affected by changes in oxygen conditions (Fig. 1A to C). A point worth stating is that our data show that the apparent increase in Hcp2 expression induced by anaerobic conditions is completely counteracted when Dnr is mutated (Fig. 1C), indicating that Dnr is a key restrictive regulator for its activation and expression. However, when the aerobic conditions were changed to anaerobic conditions, the expression of Hcp1 was reduced, but Hcp1 expression was also significantly lower in the Δ*dnr* mutant than that of the wild-type PAO1 (Fig. 1A), suggesting that the anaerobic expression of Hcp1 is regulated not only by Dnr positively but also by other factors. It remains to be refined as to what these factors are and how they are regulated.

There are 122 putative *anr* boxes (5′-TTGATNNNNATCAA-3′) in P. aeruginosa PAO1, including the *azu*, *hsiA2*, *modA*, and *dnr* genes (48). Azu is a Cu^2+^-binding protein, and Anr upregulates the expression of Azu (48, 49). Azu is also a substrate for P. aeruginosa H2-T6SS (26). Perhaps there is a cascade relationship whereby Anr indirectly enhances Azu expression by directly activating the expression of H2-T6SS. The gene promoter is a critical region for gene transcriptional regulation. By alignment to the PAO1 reference genome, the promoter regions of the *hsiA2*, *modA*, and *dnr* genes all contain a conserved *anr* box (see Fig. S3A in the supplemental material), indicating a regulatory association between them. As in mediating the uptake of MoO_4_^2−^, the secretion of ModA depends on the activation of anaerobic expression of H2-T6SS by Anr (27). These illustrate the core importance of H2-T6SS in the global regulation of downstream gene expression by Anr and extend our understanding of the relationship between bacterial H2-T6SS and anaerobiosis. There is a gene binding motif, 5′-TTGATTCGTATCAA-3′, in the promoter region of *hsiA2* (Fig. S1 and S3). We also confirmed that Anr and Dnr directly activate HsiA2 (27) (Fig. 1D and F and Fig. 2E). The presence of a homologous *anr* box in the *hcp2* promoter region has not been predicted in previous studies. Motif analysis was conducted on the MEME website (MEME version 5.4.1; https://meme-suite.org/meme/tools/meme). We also noted that the conserved binding motif is present in the promoter region of *hcp2* (5′-GTGCCACTAGTCTT-3′) (Fig. S3B), but there is no similar consensus sequence in the promoter regions of *hcp1* and *hcp3*. It has been proven that Dnr bound significantly only to *hcp2*, but not to *hcp1* or *hcp3* (Fig. 2B to D). These findings indicate that Dnr has a direct regulatory effect on *hcp2*. The results of the direct binding of Dnr to *hsiA2* and *hcp2* further show that Dnr transcriptionally regulates only H2-T6SS. Interestingly, Anr, which is upstream of Dnr, also directly activates HsiA2 and Hcp2 (27), so is there redundancy between Anr and Dnr? We overexpressed Anr in a Δ*dnr* strain. Neither Hcp2 nor HsiA2 expression was restored to some extent (Fig. S4). This suggests that the anaerobic regulation of Anr on H2-T6SS is based on the presence of Dnr. Conversely, anaerobic expression of Hcp2 was partially restored, but not at wild-type levels, by complementing Dnr in a Δ*anr* background (Fig. S4A); the expression defect of HsiA2 could not be overcome by complementing Dnr in a Δ*anr* background (Fig. S4B). These results suggest that the presence of Dnr would be more effective than the presence of Anr for Hcp2, but that full and sufficient expression requires the copresence of two transcription factors; Anr and Dnr are simultaneously indispensable for the expression of HsiA2. Therefore, whether H2-T6SS remains fully active under anaerobic conditions is not determined by a single anaerobic transcriptional regulator (Anr or Dnr), and the division of labor between Anr and Dnr is an interesting and worthwhile question for future in-depth study. All of these results imply a complex regulatory relationship between T6SS and anaerobiosis, perhaps going well beyond the involvement of Anr and Dnr.

The quick contraction of the sheath and the disassembly of the T6SS apparatus lead to the secretion of effectors from the intracellular to the extracellular compartment or across a target cell membrane (36). After initially determining that Dnr positively regulates the anaerobic protein expression of HsiA2 and Hcp2 (Fig. 1E and F), we performed comparative *in vivo* and *in vitro* analyses of the anaerobic expression of ClpV2, the second indicator of H2-T6SS function (17), and confirmed that the anaerobic activity of ClpV2 is also promoted by Dnr (Fig. 3), consistent with previous results of Anr regulation (27), suggesting the essential role of Dnr for the disassembly and the delivery function of the H2-T6SS apparatus under anaerobic conditions. On the other hand, nitrate respiration under anaerobic conditions requires the catalysis of nitrate reductase, and molybdenum is a central cofactor for the activity of dissimilatory nitrate reductase. The uptake of molybdenum ions is mediated by ModA (10, 37, 38), which depends on the secretion of H2-T6SS (27). In this work, we also demonstrated that Dnr promotes the anaerobic secretion of ModA (Fig. 5A). Neither the absence of *modA* nor that of *dnr* nor *clpV2* was sufficient to maintain normal anaerobic growth (Fig. 4). Although the loss of ClpV2 was not a restrictive determinant for the effect of anaerobic growth and nitrate reductase activity of the strain compared to ModA and Dnr, it still reduced the physiological growth and competitive advantage of the bacterium in anaerobic environments (Fig. 4 and 5B). Importantly, when we deleted *dnr* in the context of the Δ*clpV2* strain, the Δ*dnr* Δ*clpV2* mutant showed less competitive ability than the Δ*dnr* mutant. The complementation of *dnr* does not restore it to the level of the Δ*clpV2* mutant (Fig. S5), indicating that the attenuation of growth competition is also partly functionally impaired.

**FIG 6:**
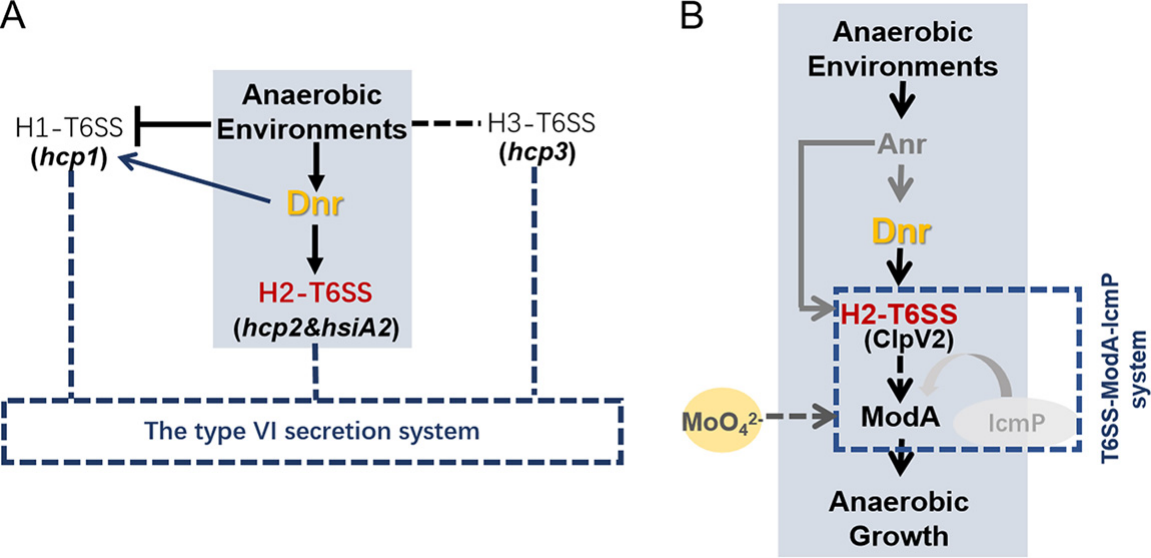
Schematic representation of the regulatory cascade involving the modulation of P. aeruginosa T6SS by the transcriptional regulator Dnr in an anaerobic environment. (A) Under anaerobic conditions, the transcriptional regulator Dnr was induced to an expression state to positively regulate the activity of H2-T6SS in response to oxidative stress. Dnr also partially activated the expression of H1-T6SS, but the anaerobic environment inhibited the transcription of H1-T6SS. There was no regulatory relationship between H3-T6SS and either oxidative stress or Dnr. Black arrowheads indicate positive regulation, the black blunt end indicates negative regulation, the black dashed line indicates irrelevant regulation, the blue arrowhead indicates noncritical positive regulation, and the blue dashed lines indicate the attributed relationship. (B) The anaerobic environment contributes to the induced activation of Dnr, whose expression is controlled by the global regulator Anr. Anr is capable of activating H2-T6SS directly or indirectly by controlling the expression of Dnr. H2-T6SS plays a core role in the T6SS-ModA-IcmP system (an active metal ion transport system) and is dependent on Dnr to activate the expression of H2-T6SS, which allows the secretion of ModA required for anaerobic growth. Black solid arrowheads indicate a positive regulatory cascade obtained in this study, the black dashed arrowhead indicates the secretory process, gray arrowheads indicate reported regulation models, and the blue dashed box indicates the active metal ion transport system.

It is well known that microaerobic environments or anaerobic conditions dominate the growth of P. aeruginosa during the chronic lung infections of CF patients, and these conditions are in turn well suitable for biofilm formation, which promotes chronic persistent bacterial infection as well as the development of antibiotic resistance (3, 50–53). Anaerobic growth has become an advantage for P. aeruginosa to adapt to the environment. The present study further complements the T6SS-mediated active metal ion transport system in anaerobic environments. Three T6SS loci (H1-, H2-, and H3-T6SS) have been identified in P. aeruginosa (17), and we identified the anaerobic expression of H2-T6SS was promoted by Dnr and anaerobiosis (Fig. 6A). The anaerobic environment contributes to the induced activation of Dnr, whose expression is controlled by a master regulator, Anr. Anr is capable of activating H2-T6SS directly or indirectly by controlling the expression of Dnr. H2-T6SS plays an important role in the T6SS-ModA-IcmP system (an active metal ion transport system) (27) and benefits from the activation of H2-T6SS expression by Dnr, which upregulates the secretion of ModA to be required for nitrate reduction and anaerobic growth competition (Fig. 6B). In addition, the maintenance of anaerobic metabolism contributes to the function of H2-T6SS to enhance the competitive advantage for anaerobic growth.

In conclusion, this study elucidates that Dnr, a specific regulator of nitrate respiration in P. aeruginosa, encourages the anaerobic expression of H2-T6SS and that this regulation is essential for ModA protein secretion, anaerobic growth, and bacterial competition. All of these findings reveal that the functional advantage of H2-T6SS in response to anaerobic induction may be a conditional environmental adaptation.

## MATERIALS AND METHODS

### Bacterial strains and growth conditions.

The bacterial strains, plasmids, and primers used in this work are listed in Tables S1 and S2 in the supplemental material. E. coli strains were cultured at 37°C in Luria-Bertani (LB) broth (1% tryptone, 0.5% yeast extract, 0.5% NaCl) and supplemented with appropriate antibiotics. P. aeruginosa (PAO1) strains were cultured in LB medium, Pseudomonas isolation agar (PIA), or M9 medium (Na_2_HPO_4_, 6g/L; KH_2_PO_4_, 3g/L; NaCl, 0.5g/L; NH_4_Cl, 1g/L; MgSO_4_, 1 mM; CaCl_2_, 0.1 mM; glucose, 0.2%) at 37°C with appropriate antibiotics when necessary. Antibiotics were added to growth media at the following concentrations: for E. coli, 25 μg/mL gentamicin (Gm), 50 μg/mL kanamycin (Kan), 100 μg/mL ampicillin (Amp), and 10 μg/mL tetracycline (Tet) in LB medium; for P. aeruginosa strain PAO1, 50 μg/mL Gm in LB medium or 150 μg/mL in PIA, and 300 μg/mL trimethoprim (Tmp) and 500 μg/mL carbenicillin (Cb) in LB medium.

P. aeruginosa cultures were performed under aerobic or anaerobic conditions (with the culture placed in a sealed AnaeroPack rectangular jar) in LB or M9 medium supplemented with 15 mM KNO_3_ as the nitrogen source (3, 4, 10, 54). Cells were harvested from bacterial cultures under two oxygen conditions during the mid-exponential phase of growth at an optical density at 600 nm (OD_600_) of 1.0.

### Construction of deletion mutants.

For gene replacement, a *sacB*-based strategy was employed as described previously (55). To construct the *dnr* deletion mutant, a 1,828-bp fragment upstream of the start codon and a 1,964-bp fragment downstream of the stop codon flanking the gene were PCR amplified and digested with EcoRI/XbaI and XbaI/HindIII, respectively. The two fragments were then ligated into pEX18Ap plasmid, which was digested with EcoRI/HindIII, resulting in pEX18Ap-*dnr*, in which the whole *dnr* open reading frame (ORF) had been deleted. The resultant plasmid was electroporated into PAO1, followed by selection for single crossovers and double crossovers (56), and the resulting Δ*dnr* mutant was created. Successful deletions were verified by PCR. The Δ*retS*, Δ*retS* Δ*clpV2*, Δ*clpV2*, and Δ*modA* mutants had been generated in the laboratory previously.

### Construction of plasmids.

To construct the complementary vector *p-dnr*, a 964 bp fragment containing the *dnr* coding regions with its promoter was PCR amplified from PAO1 genomic DNA using primers listed in Table S2, digested with BamHI/HindIII, and then ligated into a shuttle vector, pAK1900, resulting in pAK-*dnr*. The pAK-*clpV2* plasmid had been constructed in this laboratory previously. The plasmid pMS402, with a promoterless *luxABCDE* gene cluster, was used to construct the *lux* reporter strains. Both the *hcp1*, *hcp2*, *hsiA2*, and *hcp3* gene promoter regions were amplified by PCR and cloned into the pMS402, yielding pKD-*hcp1*, pKD-*hcp2*, pKD-*hcp3*, and pKD-*hsiA2*. Expression plasmids mini-CTX-*hcp2*-Flag and mini-CTX-*hsiA2*-flag were produced by mini-CTX-*lacZ* containing the entire *hcp2* and *hisA2* genes and the C-terminal 3 × Flag sequence, which had been constructed based on the previous work in the laboratory. Plasmid mini-CTX-*modA*-flag was produced by mini-CTX-*lacZ* containing the entire *modA* gene and the 3× Flag tag, which had been constructed previously. To express His_6_-tagged *dnr*, primers *dnr*-F-BamHI and *dnr*-R-HindIII were used to amplify the *dnr* gene fragment from the genomic DNA of PAO1. The PCR products were digested with BamHI/HindIII and inserted into the BamHI/HindIII sites of pET28a, resulting in plasmid pET28a-*dnr*. All constructs were sequenced to verify that no mutations had been incorporated.

### Expression and purification of Dnr protein.

Recombinant plasmid pET28a-*dnr* was transformed into E. coli BL21(DE3), and the recombinant strains were cultured in LB medium supplemented with 50 μg/mL kanamycin at 37°C overnight. Every 10 mL of revived bacterium suspension was inoculated into 1 L LB medium and grown at 37°C with continuous shaking (220 rpm) to an OD_600_ of 0.6 to ~0.8. Then, 0.5 mM isopropyl β-d-1-thiogalactopyranoside (IPTG) was added to induce expression. After incubation at 16°C for another 20 h, cells were harvested at 4°C following centrifugation at 9,500 rpm. Pellets of Dnr were resuspended and lysed in A buffer (10 mM Tris-HCl [pH 7.5], 500 mM NaCl, 10% glycerine, 1 mM dithiothreitol [DTT]) supplemented with 1% lysozyme and protease inhibitors and then broken by sonication until the suspension was translucent. The clear cell lysate was purified using a Ni-Hi Trap chelating high-performance (HP) column (GE Healthcare) equilibrated with A buffer according to the manufacturer’s instructions. The protein was eluted with 500 mM imidazole in the same A buffer using a stepwise gradient. Purified Dnr proteins were dialyzed in C buffer (10 mM Tris-HCl [pH 7.5], 200 mM NaCl, 1 mM DTT) overnight at 4°C and then concentrated to 1 mL and stored at −80°C until used. The concentration of protein was determined by using the Bradford protein assay kit (Bio-Rad) according to the manufacturer’s instructions, with bovine serum albumin (BSA) as standard.

### Electrophoretic mobility shift assays.

Various amounts of Dnr proteins were mixed with different DNA promoters in 20 μL of the gel shift loading buffer (25 mM Tris-HCI [pH 7.5], 10 mM MgCl, 1 mM DTT, 10% glycerol, 15 μg/mL sheared salmon sperm DNA). After incubation at room temperature for 15 min, aliquots of the binding reaction mixtures were then electrophoresed on 7% nondenaturing acrylamide gels in 0.5× Tris-borate-EDTA (TBE) buffer. The gels were stained by Gelstain and subjected to screening on a phosphor screen (LAS600; GE Healthcare).

### Luminescence expression assays.

The expression of *lux*-based reporters from cells grown in liquid culture was measured as counts per second (cps) of light production in a Synergy 2 plate reader (Biotek). The pKD-related plasmids were electroporated into P. aeruginosa recipient strains. Overnight cultures of bacteria were subcultured at a 2% dilution with fresh LB medium with 15 mM KNO_3_ added, and then the cells were grown under aerobic and anaerobic conditions up to an OD_600_ of ≈1.0. The cultures were added to a black 96-well plate with a transparent bottom. The bacterial luminescence value and the OD_600_ value were measured in a scanning mode in a Synergy 2 plate reader (Bio Tek), and the promoter activity was measured at a ratio of cps to OD_600_ (relative luminescence value).

### Western blot assays.

The resultant plasmid was electroporated into the PAO1 wild type or Δ*dnr* mutant. Overnight cultures of bacteria were subcultured with 2% dilution in fresh LB with 15 mM KNO_3_ under aerobic and anaerobic growth conditions as described above. The supernatant and pellets were separated by centrifugation. The bacterial supernatant was treated with 5% trichloroacetic acid (TCA) at 4°C to precipitate proteins. Precipitated proteins were collected by centrifugation and washed with acetone. Samples from equivalent numbers of bacterial cells were loaded and separated by 12% SDS-PAGE gels. The proteins were transferred onto a polyvinylidene difluoride (PVDF) membrane and hybridized with mouse monoclonal Flag and RNA polymerase (RNAP) antibodies (Sigma). The signal was detected by the use of an ECL Plus kit (Amersham Biosciences). All Western blot assays have been repeated a minimum of three times with independently derived protein samples. Representative blots are shown.

### Fluorescence microscopy.

Overnight-cultured strains harboring mini-CTX-*clpV2*-*sf*GFP vector were used for expression of the *clpV2*-*sf*GFP fusion protein. For aerobic or anaerobic growth, P. aeruginosa strains were cultured in LB supplemented with 15 mM KNO_3_ at 37°C to an OD_600_ of 1.0. Bacterial cells were harvested and resuspended in phosphate-buffered saline (PBS) and then placed on 1% agarose pads and examined immediately at room temperature. A Nikon Ti2-E inverted microscope with a perfect focus system and a CFI Plan Apo Lambda 100× oil Ph3 DM (NA 1.4) lens objective was used for imaging. Intensilight C-HGFIE (Nikon), and ET-GFP (49002, Chroma) filter sets were used to excite and filter fluorescence. NIS-Elements AR 5.20.00 was used to record and manipulate the images.

### Nitrate reductase assay.

Overnight bacterial cultures were transferred to the LB liquid medium supplemented with 15 mM KNO_3_ at a volume ratio of 1:50 and incubated at 37°C for 24 h under anaerobic conditions. The activity of nitrate reductase was determined via a diazo-coupling reaction as described previously (27, 39). Bacterial cells (1.5 mL) were aspirated into a centrifuge tube, and 25 μg/mL of chloramphenicol was added to inhibit protein synthesis. After incubation for 5 min, cells were centrifuged at 2,250 × *g*, washed twice, and resuspended in an equal volume of 50 mM phosphate buffer (pH 7.2), and the optical density at 600 nm (OD_600_) was determined using 200 μL culture. One hundred microliters of freshly prepared 0.5 mg/mL methyl viologen was mixed with 800 μL of cell suspension. Nitrate reduction was initiated by adding 100 μL of a solution containing 4 mg/mL Na_2_S_2_O_4_, 4 mg/mL NaHCO_3_, and 100 mM KNO_3_. Control reaction mixtures replaced Na_2_S_2_O_4_ with water. Reaction mixtures were incubated at room temperature for 5 min and appeared blue, and then reactions were stopped by vortexing until the solution became clear, indicating the electron donor was oxidized. One milliliter of 1% sulfanilic acid prepared in 20% HCl was added immediately to stop the reaction, and the mixture was vortexed for 15 s. One milliliter of 1.3 mg/mL *N*-(1-naphthyl) ethylenediamine dihydrochloride was added to permit the formation of a red azo dye. After incubation for 13 min, the suspension was centrifuged to pellet debris. Two hundred microliters of the clarified solution was transferred to a microtiter plate. Using a Synergy 2 plate reader (Bio Tek) in the scanning mode, the OD_540_ of the supernatant was measured to quantitate dye formation, and the OD_420_ was measured to account for absorbance due to light scattering by residual cells or cell fragments. The activity of nitrate reductase was expressed in arbitrary units, as calculated using the following formula, where the parameter *T* (minutes) represents time and the parameter *V* (milliliters) represents mixed reaction volume:

$$\text{activity\%}=100\%\times \frac{{\text{OD}}_{540}-\left(0.72\times {\text{OD}}_{420}\right)}{T\times V\times {\text{OD}}_{600}}$$

### Anaerobic growth assay.

The overnight cultures of bacteria were transferred to the LB liquid medium at a 2% dilution supplemented with 15 mM KNO_3_ and incubated under anaerobic conditions for 24 h at 37°C. The anaerobic growth of each bacterium was determined by measuring the OD_600_ using the scanning mode in a Synergy 2 plate reader (Bio Tek).

### Interbacterial growth competition assays.

Interbacterial competition assays were conducted as described previously, with minor modifications (27, 57). Briefly, overnight cultures of relevant P. aeruginosa strains (carrying the complementary vector pAK-*dnr* or the empty vector pAK1900, which indicates Cb^r^) and the E. coli competitor strain carrying the PBBR1-MCS5 vector (Gm^r^) were adjusted to OD_600_ of 1.0 with M9 medium and then mixed in 1:1 (vol/vol) of relevant P. aeruginosa versus the competitor E. coli in M9 medium containing 15 mM KNO_3_ supplemented with or without 10 μM Na_2_MoO_4_. To calculate the initial CFU ratio of relevant P. aeruginosa and competitor, 50 μL of the mixture was taken out, serially diluted, spread on LB plates containing different antibiotics, and incubated at 37°C for 12 h. For competition assays, the culture was incubated at 37°C under anaerobic conditions, and after 12 h, the mixture was serially diluted and spread on LB plates containing different antibiotics, and then the final CFU ratio was determined.

### Statistical analysis.

All experiments were performed in triplicate and independently repeated three times. Differences between means were determined through ordinary one-way analysis of variance (ANOVA) and two-way ANOVA with Dunnett’s test, with a significance level of *P* < 0.05.
