# Supplementary material for: An Important Role of the Type VI Secretion System of Pseudomonas aeruginosa Regulated by Dnr in Response to Anaerobic Environments
Source: Microbiol Spectr. 2022 Oct 27;10(6):e01533-22. doi: 10.1128/spectrum.01533-22 (PMC9769707; doi:10.1128/spectrum.01533-22)
Supplement: Supplemental file 1 — Supplemental material. Download spectrum.01533-22-s0001.pdf, PDF file, 1.2 MB [file spectrum.01533-22-s0001.pdf]

Supplemental Material

Table S1. Bacterial strains and plasmids were used in this study.

Strain or plasmid	Relevant characteristics	Source
E. coli		
DH5α	F^- φ80lacZ ΔM15 Δ(lacZYA-argF) U169 recA1 endA1 hsdR17(rk^-, mk^+) phoA supE44 thi-1 gyrA96 relA1 tonA	Stratagene
**BL21 (DE3)**	F^- ompT hsdS_B (rB^- m_B^-) gal dcm met (DE3)	Invitrogen
P. aeruginosa		
**PAO1**	Wild type	This lab
**Δ*dnr***	*dnr* deletion mutant of PAO1	This study
**Δ*modA***	*modA* deletion mutant of PAO1	This lab
**Δ*retS***	*retS* deletion mutant of PAO1	This lab
**Δ*clpV2***	*clpV2* deletion mutant of PAO1	This lab
**Δ*retS*Δ*clpV2***	*retSclpV2* deletion mutant of PAO1	This lab
**Δ*anr***	*anr* deletion mutant of PAO1	This lab
**Plasmids**		
**pET28a**	T7 *lac* promoter-operator, N-terminal His tag, Kan^r	This lab
**pEX18Ap**	$oriT^+$ $sacB^+$ gene replacement vector with mμltiple-cloning site from pUC18; Ap^r	This lab
**pAK-1900**	*E. coli-P. aeruginosa* shuttle cloning vector carrying plac upstream of MCS; Ap^r, Cb^r	This lab
**mini-CTX-*lacZ***	Integration plasmid; Tc^r	This lab
**pMS402**	Expression reporter plasmid carrying the promoterless luxCDABE gene; Kn^r, Tmp^r	This lab
**pKD-*hcp1***	pMS402 containing *hcp1* promoter region; Kn^r, Tmp^r	This study
**pKD-*hcp2***	pMS402 containing *hcp2* promoter region; Kn^r, Tmp^r	This study
**pKD-*hcp3***	pMS402 containing *hcp3* promoter region; Kn^r, Tmp^r	This study
**pKD-*hsiA2***	pMS402 containing *hsiA2* promoter region; Kn^r, Tmp^r	This study

**pET28a-*dnr***	Protein expression construct, the entire gene of *dnr* cloned in pET28a vector	This study
**pEX18Ap-*dnr***	*dnr* deletion plasmid, pEX18Ap with upstream and downstream region of *dnr*; Ap^{r}	This study
**pAK-*clpV2***	pAK1900 with the entire *clpV2* gene; Ap^{r}	This lab
**pAK-*dnr***	pAK1900 with the entire *dnr* gene; Ap^{r}	This study
**pAK-*anr***	pAK1900 with the entire *anr* gene; Ap^{r}	This lab
**mini-CTX-*hcp2*-flag**	Expression plasmid, mini-CTX-*lacZ* containing the entire *hcp2* gene and the 3x *flag* sequence; Tc^{r}	This lab
**mini-CTX-*hsiA2*-flag**	Expression plasmid, mini-CTX-*lacZ* containing the entire *hsiA2* gene and the 3x *flag* sequence; Tc^{r}	This lab
**mini-CTX-*clpV2*-*sf*GFP**	Expression plasmid, mini-CTX-*lacZ* containing the entire *clpV2* gene fused with *sf*GFP sequence; Tc^{r}	This lab
**mini-CTX-*modA*-flag**	Expression plasmid, mini-CTX-*lacZ* containing the entire *modA* gene and the 3x *flag* sequence; Tc^{r}	This lab

Table S2. Primers were used in this study.

Primer	Sequence (5'→3') [a]	Application
**pEX-*dnr*-up-S**	TATgaattcGAGCTGAACCGCGAACTGTA	Constructing *dnr*
**pEX-*dnr*-up-A**	TATtctagaAGTGCTTCGAGTGAACAGCG	deletion mutant
**pEX-*dnr*-down-S**	TAAtctagaATGCTGGGAAGGCTCGCGAT	
**pEX-*dnr*-down-A**	ATTaagcttAGGCGTTTCGCCGCTGTCTT	
**pAK-*dnr*-S**	ATTggatccCCGTGCTACGCCACCATC	Constructing *dnr*
**pAK-*dnr*-A**	TAGaagcttTCACTCGAAGCACTCCAGGCG	complemented plasmid
**pET28a-*dnr*-S**	ATAggatccATGGAATTCCAGCGCGTC	Protein cloning
**pET28a-*dnr*-A**	AATaagcttCTCGAAGCACTCCAGGCG	
**p*hcp1*-gf-S**	TGCTACTCCTTGCATTGCCAGCGCC	
**p*hcp1*-gf-A**	GGTGACGATCTCCCTATCATCGAAG	
**p*hcp2*-gf-S**	GCCTTGGCCGAAAGGCTA	
**p*hcp2*-gf-A**	GGATACGTTCCTTGTCGTTGAAAG	Electrophoretic Mobility
**p*hsiA2*-gf-S**	GGACCGCCCATCTCGATT	Shift Assay
**p*hsiA2*-gf-A**	ATGCGAGGAGAGCTTGCTC	
**p*hcp3*-gf-S**	GGGAGTCCAACGAAAATTT	
**p*hcp3*-gf-A**	GGCGGCTGACTCCGATGCAA	
**p*Control*-gf-S**	GACCTTCGCCGATGCCGA	
**p*Control*-gf-A**	AGCCGGTAGCTCGCTCCG	
**pKD-*hcp1*-S**	TAActcgagAGCGGCATTGCTACTCCTTG	
**pKD-*hcp1*-A**	ATTggatccTACATCCAGCACGGTGACGA	
**pKD-*hcp2*-S**	AAActcgagAACGCAACCTGATCGGCAGC	
**pKD-*hcp2*-A**	AATggatccCTTGGTGCCGGTGATGGACA	Luminescence
**pKD-*hsiA2*-S**	TATctcgagGGTATGTGGGGATGCTGGA	Expression Assay
**pKD-*hsiA2*-A**	TAGggatccATGCGAGGAGAGCTTGCTC	
**pKD-*hcp3*-S**	TATctcgagCCGTGCTTCATGGGAGTCCA	
**pKD-*hcp3*-A**	ATTggatccTGCTGCGTACTCTCGGCCAT	

[a] Restriction site is displayed in lowercase.

hsiA2 promoter (*PA1656*)

GGACCGCCCATCTCGATTAATTTCTCTAACAATTCCCGTGCCAGTCTTTTCGTGGCTCTTTTAATCAATCTCA
TTCAATGGCTTATTGTTATCCATGTCTCATTCTGAAAAGAAATATAACAAGTGAATTGAGCAAGAAGTTGCGC
AGTGCTGTGCAACTTTTTGCGCAGTAGTGTTCAGGCCAGTATTTTCAAGGGCTGTGGACTGTTATTGGGGATG
TTTCTTGGTTTTCATTGTGCAACTTCTTGCACAATCGCTATTACGCTCTAAATCAGCTGTTGGTCCGCTTGGA
AGGCCTATGTTTCGAGGCTCTCCGGAGAAATCCATACTGCCTGCCTTATTTTTTATAAATTGTTAACTACCTG
TTTTGGTAGGGTTTTCCAAATGGCCATCAATGTCGTTATGATGGCTATAGCCAAGCCGTAAAGGGCATGGACG
CAGACGCCTGACGTTTGGCTGAAAATCTTTCA**TTGATTCGTATCAA**TGAATAGGAAAATTCCCAAAGAGGGAA
TGTCCTGTTGCTTGCCAGTTTTTATGTTGTTTTCATGGAAAAGGCAAAGAGCTCTTCAGACTATTGGGATTTT
CGTTCGGTTTTTATGCCAACTAGTTGAATTGTTAAGATATTCATTGGCGCACATGGTGTGCATTGAAGGGTTA
GG**ATG**ACCTATTC**GAGCAAGCTCTCCTCGCAT**

hcp1 promoter (*PA0085*)

TGCTACTCCTTGCATTGCCAGCGCCCGGCTCTTCCAGGCGCTCACCCCCGTGCCCGAGGGATTTCGGTTCGCA
TCCAGGCTGGCCCGACGGCTTTCCGGCGTTTCGTCGCCTCGGGAAGACACATCGGGTGATACCGGATTATAAA
AGACTAATCCTACGTCTTTTAAAGATTTGTCCGATCTGCGGTGTCATGCAACTAGTGGCGTTGTGCCTTCCTT
GACAAGCCTTGCGGCATGCCACAAGAATTTTGCCAACCTTTCGAGTCATCCAATATTCATCAATGGCTCCTAC
AAGATCCGACGGAGTGGATCTTACAAGCGAAGGTGAGGGCCGCGCAGCGG**CTTCGATGATAGGGAGATCGTCA
CC**

hcp2 promoter (*PA1512*)

GCCTTGGCCGAAAGGCTAAGAACCGCACCTCCGGTTCCCCGCAGCAGAACAACCCTGCTGGCCCTACCCCAAA
AGCCCTCTCCATAGAGGGCTTTTGGCCATAACTGGCCATATCTTTCAGCCCCCAACCTCTCCGATAACTCACT
TTCCTTCACCCTGGGCCGATGGAACGAAACCGTGCGGCGCTATCTTCGCCTACCTTGCCAATGAAGAACTGTG
CAACTTTTTGGTTGATAACGTTGTGGATAGAACGCTAGGGAACAAATACCCAAAGGGCTCCGAGAGAGTGCGC
AACTTTTTGCAAGCGGTGCGCAAAAAGTTGCGCAATTTTCAGTGTTCTTCCCATGAGAATCGCCAGCGAATTT
TTGGCTTCCCCCAATAACGCCTTGATGGAAAAGAGTTTC**AAGACTAGTGGCAC**GGTTCTTGTCTAGCACCGAT
GCCCCCGGCATATCGCCTGGGCA**CTTTCAACGACAAGGAACGTATCCATG**GCAACCC

hcp3 promoter (*PA2367*)

GGGAGTCCAACGAAAATTTTATTTTGCAAATCCATAGACCTGTTCTAGCGTCTTTTGTAGTTCGTCGCCAGTG
ACGGACCAGAGGGATCGCCAAGGACAGGTTCTGCGCCGTTTTCCCTGTCATGCCTGTGATCCAGCAAGGGACT
GCGATGATCAGGGCATCCCCCCGATGCATTGCTCCCCCACCA**TTGCATCGGAGTCAGCCGCC**

FIG S1 The promoter regions of T6SS genes used in EMSA assays. Underlined regions indicate the position of the primers for promoter sequence synthesis; rectangular box indicates the binding motif in the promoter region; the start codons (ATG) of *hsiA2* and *hcp2* are indicated by thick lines.

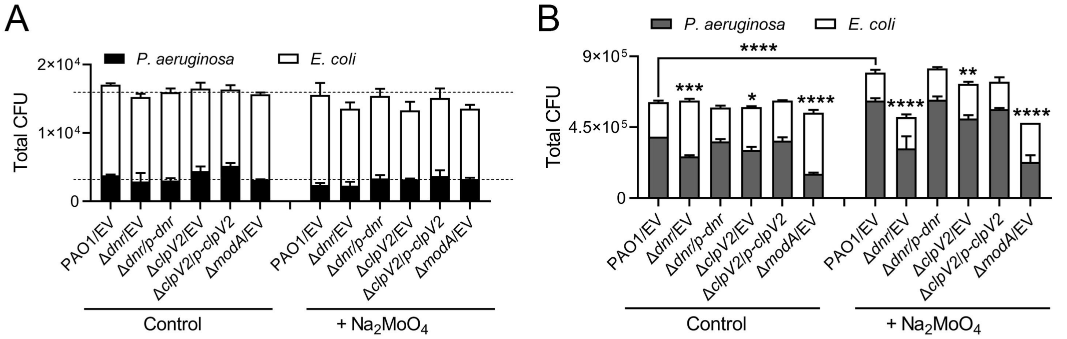

FIG S2 Interbacterial growth competition assays between *P. aeruginosa* and *E. coli*. Competition between the indicated strains was examined in M9 containing 15mM KNO_3 at 37°C for 12h under anaerobic conditions. Quantification of CFU (A) before (initial) and (B) after (final) growth competition assays between the indicated organisms. Data shown are the average of three independent experiments; error bars indicate the SD from three independent experiments. Statistical significance was calculated using one-way ANOVA Dunnett′s multiple comparison test; ****, p<0.0001; ***, p<0.001; **, p<0.01; *, p<0.05.

A

***p-hsiA2* (*PA1656*)** [+ strand] ATG distance: 176bp_From 1803451 to 1803626 bp

5′-TTCA**TTGATTCGTATCAA**TGAATAGGAA...........TGAAGGGTTAGG**ATG**ACCT-***3′***

***p-modA* (*PA1863*)** [- strand] ATG distance: 96bp_From 2023166 to 2023262 bp

5′-CGAC**TTGCGCCGGATCAA**TTTCCGCGTA...........CAGGAGCACTCC**ATG**ACCA-***3′***

***p-dnr* (*PA0527*)** [- strand] ATG distance: 62bp_From 586663 to 586724 bp

5′-GAGC**TTGCCGTGCGTCAA**GATGATCTGC...........AGCCTTCCCAGC**ATG**GAAT-***3′***

***p-hcp2* (*PA1512*)** [- strand] ATG distance: 68bp_From 1644725 to 1644792 bp

5′-TTTC**AAGACTAGTGGCAC**GGTTCTTGTC...........AGGAACGTATCC**ATG**GCAA-***3′***

B

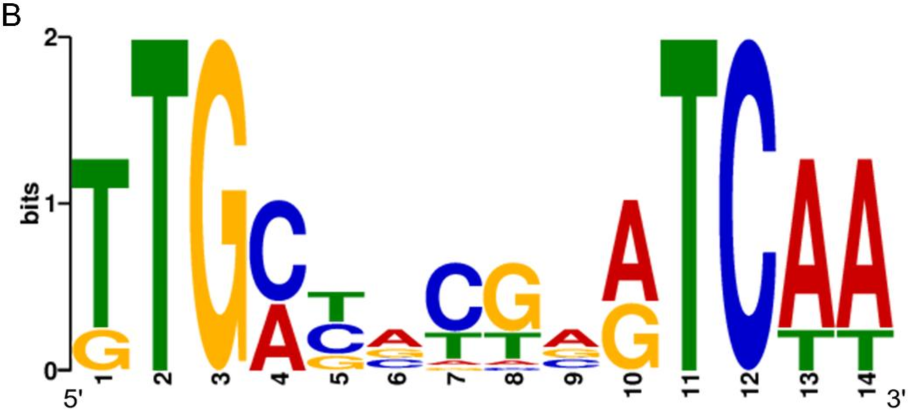

FIG S3 (A) The promoter regions of *hsiA2*, *modA*, *dnr*, and *hcp2* contain the

conserved *anr*-box. The rectangular box indicates the homologous sequence of *anr*-box in the promoter region, and the start codon (ATG) of the gene is indicated by a thick line. The middle portion of the promoter sequence has been omitted with the ellipses. (B) MEME motif analysis of consensus sequences. Numbers on the x axis indicate base number.

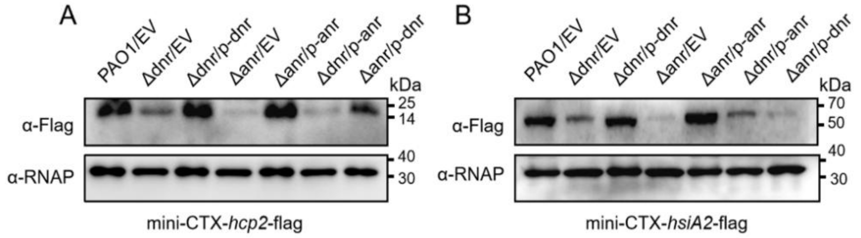

FIG S4 Dnr and Anr activate the expression of H2-T6SS under anaerobic conditions. Western blot analysis of wild-type *P. aeruginosa*, the Δ*dnr* mutant, the Δ*anr* mutant, and the complemented strains harboring *hcp2* (A) or *hsiA2* (B) flag-probed was cultured under aerobic or anaerobic conditions to OD600=1.0 in LB containing 15mM KNO_3. Similar results were obtained from three independent experiments, and the data shown are from one representative experiment.

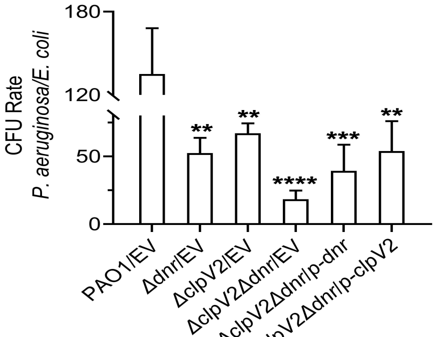

FIG S5 Dnr and ClpV2 are indispensable for interbacterial growth competition in anaerobic environments. Competition between the indicated strains was examined in M9 containing 15mM KNO_3 at 37℃ for 12h under anaerobic conditions. The CFU ratio of the relevant *P. aeruginosa* strains versus the competitors is plotted. Data shown are the average of three independent experiments; error bars indicate the SD from three independent experiments. Statistical significance was calculated using one-way ANOVA Dunnett′s multiple comparison test; ****, p<0.0001; ***, p<0.001;

**, $p<0.01$

clpv2-sfGFP foci.mp4

Video S1 Deletion of *dnr* reduces H2-T6SS assembly under anaerobic conditions, related to Figure 3.
